# Effects of Outcome Predictability on Human Learning

**DOI:** 10.3389/fpsyg.2017.00511

**Published:** 2017-04-05

**Authors:** Oren Griffiths, Anna Thorwart

**Affiliations:** ^1^School of Psychology, University of New South WalesSydney, NSW, Australia; ^2^Department of Psychology, Philipps-UniversitätMarburg, Germany

**Keywords:** contingency learning, human predictive learning, associability, cue-outcome association, outcome predictability, cue predictiveness

## Selectivity in cue-outcome learning

Cue-outcome learning is a cornerstone of intelligent action. Learning that a stimulus (the cue) can be used to predict a second event (the outcome) affords adaptive decision making. If an animal can use environmental cues to predict the presence of food (an appetitive outcome) or a predator (an aversive outcome), then it can potentially act to maximize the likelihood of the former and minimize the latter. This capacity also allows for the development of complex associative webs of knowledge. For example, it allows humans to associate a visual icon with an auditory phoneme (and thus read), or to connect new information with existing knowledge. These are fundamental capacities of intelligent agents.

The capacity to learn cue-outcome mental associations is crucial. However, forming mental associations may be cognitively costly, so it is important to be selective about which associations are learned, and thus to prioritize which stimuli gain access to the learning process. One way to achieve this efficiency is to leverage prior knowledge to focus primarily upon those events (cues and outcomes) that are most likely to be meaningfully associated (e.g., Mackintosh, 1975; Le Pelley, 2004, 2010; Mitchell and Le Pelley, 2010; Esber and Haselgrove, 2011).

There are three logically distinguishable components of a person's prior associative knowledge that could be used to guide the selectivity of subsequent cue-outcome learning. First, there is the knowledge about specific cue-outcome associations that have already been learned (represented as associative strength, or V). Second, there is knowledge about the cueing stimuli themselves (represented as cue associability, or α). Finally, people could use prior knowledge about the outcome stimuli. The influence of the first two forms of associative knowledge in guiding subsequent learning has been extensively investigated. However, the influence of the third form (i.e., information about outcome stimuli) has been largely overlooked in the learning literature. We argue herein that this oversight of the potential role of outcome predictability in shaping learning offers fertile new ground for the science of learning. Before making this case, we first briefly note how associative strength and cue associability have been shown to guide learning.

## Associative strength modulates subsequent learning

There is a vast and detailed literature showing that the associative strength of cue-outcome relationships shapes the manner in which subsequent cue-outcome learning takes place. Perhaps the clearest example of this is the observation that learning curves are negatively accelerated (e.g., Rescorla and Wagner, 1972). That is, when little is known about a cue's relationship with an outcome, the person's knowledge about this association (V) increases rapidly. However, as more is known about that specific cue-outcome association, the rate of new learning about that association decreases, and eventually an asymptotic value for the associative strength is reached. In addition, learning about other associations can be impaired too, as demonstrated in the blocking effect (Kamin, 1969), perhaps the most widely studied learning phenomenon. An initial cue (A) is paired with an outcome, but is withheld for a control group. Then for both groups, a new cue (B) is shown alongside the pre-trained cue (A) and they are both followed by the outcome. Those that had already learned an association between A and the outcome fail to associate (the redundant) cue B with the outcome, relative to the control group that did not receive pre-training. The activation of the already existing cue-outcome association by its cue A blocks learning about cue B's new association with the same outcome (see superconditioning for the opposite effect; Rescorla, 1971; Wagner, 1971).

## Cue associability modulates learning

The second source of knowledge that is well-known to guide the selectivity of subsequent cue-outcome learning is the associability of the cue. Several authors have argued that animals learn which cues are valid predictors of outcomes, and this knowledge shapes how readily those cues become associated with outcomes in the future (see LePelley et al., 2016 for a review). Specifically, Mackintosh (1975) suggested that cues that are good predictors of outcomes are more *associable*; they more readily enter into associations with outcomes in future. Empirical evidence generally supports this claim (but see Pearce and Hall, 1980; Griffiths et al., 2011). People (and other animals) readily learn which cues are valid predictors, and this cue-specific learned information guides subsequent selectivity in cue-outcome learning (Le Pelley, 2004). Perhaps the clearest example of this effect is the “*Learned Predictiveness*” effect (Le Pelley and McLaren, 2003), whereby cues shown to be previously predictive of important events are more rapidly learned about subsequently.

## Does outcome predictability modulate learning?

The third possibility is that acquired, specific knowledge about outcome stimuli may bias subsequent cue-outcome learning. While it is well-known that physical properties of the outcome influence the speed of learning (Annau and Kamin, 1961), we suggest that people may learn about an abstract property of outcomes – their “predictability”– and that this modulates the formation of subsequent cue-outcome associations involving those same outcome events. Notably, traditional formal models are silent as to the possibility of such a “Outcome Predictability” effect, whereas the effects of prior cue-outcome learning and learned cue associability (see above) are both well-predicted by these models.

However, there is now growing empirical support for the hypothesis that people encode and use information about outcome predictability to guide learning. The first empirical indications that the experienced (un)predictability of an outcome might impact upon subsequent learning comes from the *learned irrelevance* and *learned helplessness* paradigms (e.g., Mackintosh, 1973; Baker, 1976; Overmier and Wielkiewicz, 1983). However, many of these findings were adequately explained by a variation of the blocking effect, termed “context blocking” (Baker et al., 1981). Even if no distinct cue with an established association (such as cue A in the earlier example) is present during subsequent learning, the diffuse and ever present contextual cues will activate their cue-outcome associations and may block new cue-outcome learning. Therefore, both effects may also be explained as the product of prior cue-outcome associations, and need not demonstrate an effect of prior learning about the unpredictability of the outcome itself.

More recently, Griffiths et al. (2015) demonstrated that people learn novel cue-outcome associations more rapidly if those associations involved outcomes that had previously been shown to be predictable, as compared to otherwise equivalent, novel cue-outcome associations involving outcomes that were previously shown to be unpredictable. Moreover, this effect is not readily attributed to context blocking (but see Liu et al., under review). In their procedure, people were tasked with learning which foods a fictional patient, Mr. X, was allergic to. They were shown the meals Mr. X ate on different days, and whether or not he had a reaction to that meal. In the first stage, Mr. X ate only vegetables, and sometimes experienced stomach (nausea, cramping) or skin (itching, swelling) reactions. Stomach reactions were predictable on the basis of vegetables ingested, but skin reactions were not. Then, in a second stage, Mr. X ate only fruits, and both stomach and skin reactions were predictable on the basis of the fruits ingested. Despite both types of allergic reaction (outcome events) being predictable in the second phase, people learned more rapidly about the associations between fruits and the previously predictable (stomach) reactions than between fruits and the previously unpredictable (skin) reactions. This bias toward learning associations involving previously predictable outcomes has since been replicated by two independent research groups using the same allergy task (Thorwart et al., in preparation), in a serial reaction time task (Quigley et al., under review) and in a human goal-tracking task (Liu et al., under review).

## Possible mechanisms

**Figure 1:**
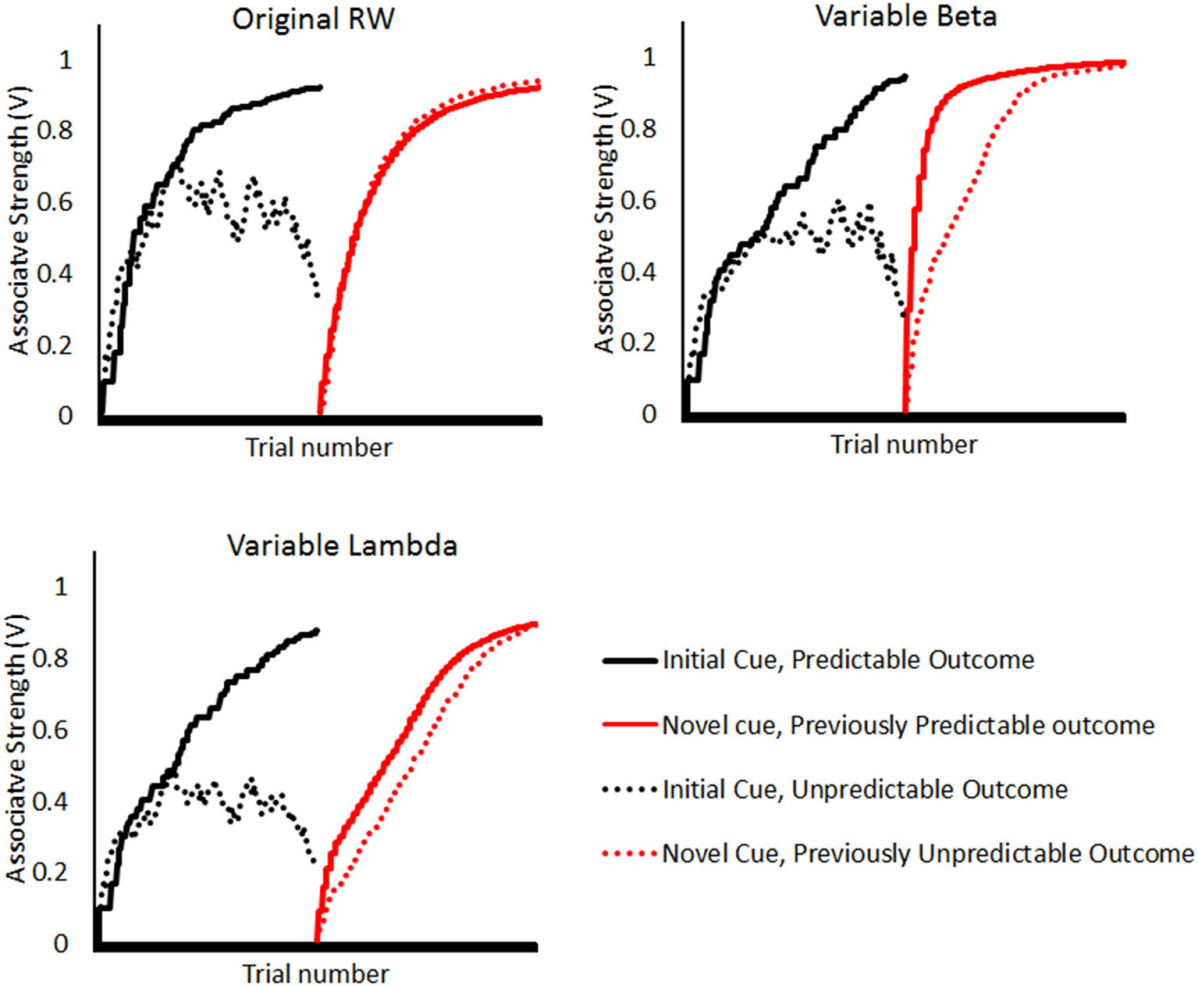
**Simulations of the Rescorla-Wagner model in either its original form (upper left panel), or in which beta (upper right panel) or lambda (lower left panel) are allowed to vary with experience**. The simulation consisted of a first stage (black lines) in which one outcome was trained to be 100% predictable (solid black lines) and one outcome trained to be only 50% predictable (broken black lines). Then in the second stage (red lines), a novel cue was reliably (i.e., on 100% of presentations) followed by the previously predictable outcome (solid red line) and a second novel cue was reliably followed by the previously unpredictable outcome (broken red line). A Learned Predictability effect is demonstrated by more rapid ascent of the solid red line than the broken red line. This can be seen in only those simulations that allow parameters associated with the outcome stimulus (lambda and beta) to vary. (In the Variable Beta and Lambda models, these parameters decreased by 10% on each trial in which the summed error term, which captures the prediction error, exceeded a threshold: 0.2 for lambda model, 0.5 for beta model. Thus, both parameters stayed high for predictable outcomes with on average small predictions errors but decreased for unpredictable outcomes with an on average larger prediction error. All other parameters, including the provision of an implied contextual predictor, were held constant across simulations).

Rescorla and Wagner's (1972) model (RW) is often viewed as the quintessential associative model of predictive learning. Using four psychologically plausible variables, it describes how learned associations between cues and outcomes change with experience. Two of these variables, associative strength (*V*) and cue associability (α) are well-known to affect cue-outcome learning. An interesting theoretical question posed by the Outcome Predictability effect is whether this effect is explicable in terms of the two variables related to the outcome stimuli, the outcome associability β or the outcome efficacy λ. Although both variables are typically interpreted as fixed properties of the outcome (depending on its physical salience or intensity, respectively), it is possible to simulate what would happen if they were allowed to vary as a product of experienced unpredictability. In Figure 1, we describe simulations of models in which previously unpredictable events either lose associability (β) or efficacy (λ) as each of the parameters is made dependent on the prediction error (see also below and Figure 1 for details). An Outcome Predictability effect is evident by the more rapid ascent of the solid red line than the broken red line in both simulations but not the original RW model. More importantly, a more thorough consideration of how these parameters may be influenced by unpredictability, and then subsequently influence learning, could elicit important, novel hypotheses for the field.

### Outcome associability (β)

Perhaps the most elegant possibility is that outcome predictability operates similarly to the well-known effects of learned predictiveness on a cue's associability. Mackintosh's (1975) model of cue associability states that associations are formed more rapidly when they involve cues which are known to be valid predictors. Perhaps an analogous process holds for outcome predictability too, whereby the outcome associability β varies proportionally to the summed prediction error on previous trials, so that recently poorly predicted outcomes decline in associability (Figure 1). This can be tested by considering whether the known properties of cue associability effects also apply to Outcome Predictability effects. For example, cue associability effects tend to involve overt attentional biases (e.g., LePelley et al., 2016) and are reduced in people with selective attention deficits (e.g., in schizophrenia, Morris et al., 2013). A second line of enquiry concerns whether cue associability and outcome predictability effects interact. If these two effects are essentially the same effect applied to different stimuli (cues and outcomes, respectively), then one might expect an additive interaction between cue-associability and outcome predictability manipulations. Initial data are not generally supportive of these hypotheses (Griffiths et al., 2015; Thorwart et al., in preparation), but given the paucity of extant research, it remains too early to exclude this possibility.

### Efficacy of outcome (λ)

Alternatively, the repeated experience of failure to predict an outcome (under partial reinforcement) may selectively reduce the efficacy of an outcome in driving learning (represented as λ). If the outcome efficacy λ decreased with continued unpredictability (specifically, summed error above a threshold value), this is sufficient to elicit an Outcome Predictability effect (Figure 1). This “devaluation” account could be considered a stimulus-specific refinement of the motivational explanation of the classic Learned Helplessness paradigm, which suggested that people become demotivated and cease learning when they experience repeated failure experiences, such as failing to predict an inherently unpredictable event. This hypothesis can be relatively straightforwardly tested in people by using appetitive (or aversive) outcome stimuli, such as monetary rewards (or aversive noise events), and modulating the value of these outcomes across the experiment. If learned predictability is a product of outcome devaluation (not learning about unpredictability), then increasing the value of that stimulus, or decreasing the cost of errors, should re-motivate people and remove the effect. Conversely, decreasing the outcome value or increasing the cost of errors should amplify the effect.

### Higher-order reasoning

As suggested by Griffiths et al. (2015), it is also possible that Outcome Predictability effects are the product of participants reasoning about the causal properties of cues and outcomes. Specifically, people may have assumed that predictability is a fixed property of the outcome event. Thus, even if they learned the contingencies between all cues and all outcomes during training, they may nevertheless have selectively discounted the observed contingencies that involved the previously unpredictable outcome because they assumed it must have been coincidental (or non-causal, at least; Thorwart and Livesey, 2016). This can be tested by manipulating instructions or placing constraints on processing resources during learning. If the effect is primarily an effect of declarative reasoning, then it ought to be most evident when people are given instructions consistent with the assumption of continued predictability, and when they are given adequate time to reason (see e.g., Mitchell et al., 2012; Shone et al., 2015 for examples in the domain of cue associability). Conversely, the effect should be minimized outside of these situations.

## Conclusion and implications

We have argued that the properties of the outcome are an important aspect of associative knowledge that guides selectivity in learning, akin to the effects of cue-associability and cue-outcome associations. We have identified (and simulated) two mechanisms whereby such effects could occur within the gold-standard RW model of associative learning, and one without (declarative reasoning). Whichever of these mechanisms best account for the effect, the existence of the effect itself offers potential insights into other learning effects. One example is the partial-reinforcement extinction effect (e.g., Haselgrove et al., 2004), whereby people are slow to learn about the absence of an outcome that was previously shown in a partial reinforcement arrangement (i.e., was unpredictable). This mainstay of the animal conditioning literature is consistent with, and indeed may be an instance of, the Outcome Predictability effect. A second possibility is that outcome predictability may modulate outcome-mediated biases in action selection and attention (e.g., Gozli et al., 2014; Gozli and Ansorge, 2016), whereby the presence of stimuli that resemble the sensory consequences of an action (i.e. the “outcome”) affects the speed with which that action is subsequently performed. One might expect the control exerted by these outcome stimuli to be dependent upon their prior predictability. This hypothesis remains to be tested.

## Author contributions

OG and AT were equally responsible for the conception, drafting and revising of the paper.

## Funding

AT contribution was supported by Project TH 1923/1-1 awarded by the German Research Foundation (DFG). OG contribution was supported by an Australian Research Council (ARC) Discovery Early Career Research Award (DE150100667).

### Conflict of interest statement

The authors declare that the research was conducted in the absence of any commercial or financial relationships that could be construed as a potential conflict of interest.

## References

[B1] Annau Z. Kamin L. J. (1961). The conditioned emotional response as a function of intensity of the US. J. Comp. Physiol. Psychol. 54, 428. 10.1037/h0042199 13683658

[B2] Baker A. G. (1976). Learned irrelevance and learned helplessness: rats learn that stimuli, reinforcers, and responses are uncorrelated. J. Exp. Psychol. Anim. Behav. Process. 2, 130–141.

[B3] Baker A. G. Mercier P. Gabel J. Baker P. A. (1981). Contextual conditioning and the US preexposure effect in conditioned fear. J. Exp. Psychol. Anim. Behav. Process. 7, 109. 7241051 10.1037//0097-7403.7.2.109

[B4] Esber G. R. Haselgrove M. (2011). Reconciling the influence of predictiveness and uncertainty on stimulus salience: a model of attention in associative learning. Proc. R. Soc. B 278, 2553–2561. 10.1098/rspb.2011.0836 21653585 PMC3136838

[B5] Gozli D. G. Ansorge U. (2016). Action selection as a guide for visual attention. Vis. Cogn. 24, 38–50. 10.1080/13506285.2016.1176095

[B6] Gozli D. G. Moskowitz J. B. Pratt J. (2014). Visual attention to features by associative learning. Cognition 133, 488–501. 10.1016/j.cognition.2014.07.014 25173722

[B7] Griffiths O. Johnson A. M. Mitchell C. J. (2011). Negative transfer in human associative learning. Psychol. Sci. 22, 1198–1204. 10.1177/0956797611419305 21859928

[B8] Griffiths O. Mitchell C. J. Bethmont A. Lovibond P. F. (2015). Outcome predictability biases learning. J. Exp. Psychol. Anim. Learn. Cogn. 41, 1–17. 10.1037/xan0000042 25706542

[B9] Haselgrove M. Aydin A. Pearce J. M. (2004). A partial reinforcement extinction effect despite equal rates of reinforcement during pavlovian conditioning. J. Exp. Psychol. Anim. Behav. Process. 30, 240–250. 10.1037/0097-7403.30.3.240 15279514

[B10] Kamin L. J. (1969). Predictability, surprise, attention and conditioning, in Punishment and Aversive Behavior, eds Campbell B. A. Church R. M.(New York, NY: Appleton-Century-Crofts), 279–296.

[B11] Le Pelley M. E. (2004). The role of associative history in models of associative learning: a selective review and a hybrid model. Q. J. Exp. Psychol. 57B, 193–243. 10.1080/02724990344000141 15204108

[B12] Le Pelley M. E. (2010). The hybrid modeling approach to conditioning, in Computational Models of Conditioning, ed Schmajuk N. A.(Cambridge: Cambridge University Press), 71–107.

[B13] Le Pelley M. E. McLaren I. P. L. (2003). Learned associability and associative change in human causal learning. Q. J. Exp. Psychol. 56B, 68–79. 10.1080/02724990244000179 12623538

[B14] LePelley M. E. Mitchell C. J. Beesley T. George D. N. Wills A. J. (2016). Attention and associative learning in humans: an integrative review. Psychol. Bull. 142, 1111–1140. 10.1037/bul0000064 27504933

[B15] Mackintosh N. J. (1973). Stimulus selection: Learning to ignore stimuli that predict no change in reinforcement, in Constraints of Learning, eds Hinde R. A. Hinde L. S.(London: Academic Press), 75–96.

[B16] Mackintosh N. J. (1975). A theory of attention: variations in the associability of stimuli with reinforcement. Psychol. Rev. 82, 276–298. 10.1037/h0076778

[B17] Mitchell C. J. Griffiths O. Seetoo J. Lovibond P. F. (2012). Attentional mechanisms in learned predictiveness. J. Exp. Psychol. Anim. Behav. Process. 38, 191. 10.1037/a0027385 22369199

[B18] Mitchell C. J. Le Pelley M. E. (Eds.). (2010). Attention and Associative Learning: From Brain to Behaviour. Oxford: Oxford University Press.

[B19] Morris R. Griffiths O. Le Pelley M. E. Weickert T. (2013). Attention to irrelevant cues is related to positive symptoms in schizophrenia. Schizophr. Bull. 39, 572–582. 10.1093/schbul/sbr192 22267535 PMC3627774

[B20] Overmier J. Wielkiewicz R. M. (1983). On unpredictability as a causal factor in “learned helplessness”. Learn. Motiv. 14, 324–337. 10.1016/0023-9690(83)90020-6

[B21] Pearce J. M. Hall G. (1980). A model for Pavlovian learning: variations in the effectiveness of conditioned but not of unconditioned stimuli. Psychol. Rev. 87:532. 10.1037/0033-295X.87.6.532 7443916

[B22] Rescorla R. A. (1971). Variation in the effectiveness of reinforcement and nonreinforcement following prior inhibitory conditioning. Learn. Motiv. 2, 113–123. 10.1016/0023-9690(71)90002-6

[B23] Rescorla R. A. Wagner A. R. (1972). A theory of Pavlovian conditioning: Variations in the effectiveness of reinforcement and non-reinforcement, in Classical Conditioning II: Current Research and Theory, eds Black A. H. Prokasy W. F.(New York, NY: Appleton-Century-Crofts), 64–99.

[B24] Shone L. T. Harris I. M. Livesey E. J. (2015). Automaticity and cognitive control in the learned predictiveness effect. J. Exp. Psychol. Anim. Learn. Cogn. 41, 18–31. 10.1037/xan0000047 25706543

[B25] Thorwart A. Livesey E. J. (2016). Three ways that non-associative knowledge may affect associative learning processes. Front. Psychol. 7:2024. 10.3389/fpsyg.2016.02024 28082943 PMC5186804

[B26] Wagner A. R. (1971). Elementary associations, in Essays in Neobehaviorism: A Memorial Volume to Kenneth W. Spence, eds Kendler H. H. Spence J. T.(New York, NY: Appleton-Century-Crofts), 187–213.

